# Long-term follow-up of functional electromyostimulation–augmented home exercise after primary reverse total shoulder arthroplasty: a prospective longitudinal assessment of the original intervention cohort

**DOI:** 10.1016/j.jseint.2026.101744

**Published:** 2026-05-25

**Authors:** Thomas Wittmann, Sandra Krawczyk, Patric Raiss

**Affiliations:** a Department of Orthopaedics and Trauma Surgery, Musculoskeletal UniversityCenter Munich (MUM), LMU University Hospital, Munich, Germany; b Department for Shoulder Surgery OCM Clinic, Munich, Germany; c Medical Faculty Heidelberg, Heidelberg University, Heidelberg, Germany

**Keywords:** rTSA, Functional electrical stimulation, Long-term follow-up, Constant Score, Rehabilitation, Deltoid strengthening

## Abstract

**Background:**

Reverse total shoulder arthroplasty (rTSA) reliably reduces pain and restores function, but shoulder function typically plateaus within the first post-operative year and gradually declines thereafter. Motion-triggered functional electromyostimulation (FES) enhances muscle activation and has shown short-term functional benefits in patients after rTSA. The long-term durability of these effects remains unclear, though sustained improvements would increase the clinical value of this time-limited intervention. Therefore, the aim of this study was to evaluate short-term outcomes after at least six months after completion of a FES program and compare them with baseline and immediate postintervention results.

**Methods:**

Ten of the initially 14 patients previously enrolled in the intervention arm of a prospective randomized controlled trial were re-evaluated after at least six months after completing a 6-week home-based, deltoid-focused FES program initiated 1 year after primary rTSA. Clinical outcomes included Constant Score (primary outcome), Subjective Shoulder Value, active range of motion, deltoid strength, and scapular kinematics assessed using identical methods to the previous randomized controlled trial.

**Results:**

Ten participants (71%) completed the follow-up (FU) at a median of 18 months (range, 6-24 months) after the intervention. The mean Constant Score improved from 65.7 ± 13.2 at baseline to 79.5 ± 7.9 postintervention (*P* < .001) and remained significantly elevated at FU (84.0 ± 4.2, *P* < .001 vs. baseline; *P* = .09 vs. post). Subjective Shoulder Value increased from 70.7 ± 16.9% to 91.1 ± 10.0% postintervention and was maintained at 92.0 ± 7.9% at FU (*P* < .001 vs. baseline). Abduction range of motion and deltoid strength showed additional long-term gains, whereas no decline was observed in any outcome parameter. No device-related complications occurred.

**Conclusion:**

In this single-arm FU, functional outcomes that improved after the FES-augmented program were maintained at mid-term reassessment. Because the control group was not re-evaluated, treatment-specific durability cannot be inferred. No deterioration was observed after discontinuation of device use. Larger controlled studies with long-term comparative FU are required before conclusions regarding treatment-specific benefit can be drawn.

Reverse total shoulder arthroplasty (rTSA) reliably reduces pain and restores shoulder function in patients with cuff tear arthropathy, massive-cuff tear, and primary osteoarthritis.^6^^,^^8^ The procedure consistently relieves pain and restores function by compensating for deficient rotator cuff through deltoid recruitment.^2^^,^^13^ Various standard post-operative rehabilitation protocols following rTSA are designed to restore glenohumeral joint mobility in the early phase and to optimize deltoid muscle recruitment during subsequent stages of recovery.^4^^,^^5^^,^^10^^,^^11^ However, despite reliable early gains, functional recovery after rTSA typically plateaus within the first post-operative year.^15^^,^^17^ Longer-term studies even indicate a gradual decline thereafter, resembling the natural aging process of the shoulder.^7^^,^^15^ To enhance shoulder function during the plateau phase and potentially prevent the subsequent gradual decline, additional interventions may contribute to the long-term preservation of function in patients after rTSA.^18^^,^^20^

The newly developed motion-triggered functional electromyostimulation (FES) enhances muscle stimulation during exercises and can modify complex movement patterns by adapting muscle recruitment.^12^^,^^20^ This concept has already demonstrated positive effects in patients with scapulothoracic dyskinesia and functional posterior shoulder instability.^12^ In the parent randomized-controlled trial, we reported that a 6-week, home-based, motion-triggered FES program designed for rTSA rehabilitation approximately 1 year after surgery resulted in a significant improvement in Constant Score (CS), Subjective Shoulder Value (SSV), and abduction range of motion (ROM) in the plateau phase of shoulder function, with a significant difference compared with a matched home-exercise control group without FES.^20^ Despite these encouraging results, the durability of the observed improvements over longer intervals remains unclear. Demonstrating a long-term effect is clinically relevant, as a time-limited adjunct like FES offers a greater therapeutic value if functional gains are sustained after discontinuation of device use. Therefore, the aim of this study was to evaluate the short-term clinical outcomes in the original intervention group from the parent randomized trial by re-evaluating the same cohort using identical outcome measures and methods at least six months after completion of the 6-week FES-augmented home exercise program and to compare these short-term values with the previously reported baseline and immediate postintervention results. We hypothesized that the short-term improvements in CS are preserved at short-term follow-up (FU). For the present FU, only the original intervention cohort was re-evaluated. The control group was not available for re-evaluation. Therefore, this analysis is descriptive and cannot attribute maintained outcomes specifically to FES.

## Materials and methods

### Study design

This longitudinal single-arm FU study evaluated clinical outcomes of the intervention group from our previously published prospective randomized controlled trial.^20^ In the parent trial, patients who had undergone primary rTSA for primary osteoarthritis, cuff tear arthropathy, or massive cuff tear using a single implant system (Ascend Flex, Stryker Inc.) by a single specialized shoulder surgeon (P.R.) and completed an additional standardized rehabilitation protocol. With a minimum of 12 months after surgery, participants were randomized to a home-based rehabilitation program with or without additional motion-triggered FES (Shoulder Pacemaker, Alyve Medical, Inc). Outcome assessments were performed pre- and postintervention in the intervention and control group and the results were statistically analyzed.

For the re-evaluation in the longer FU of the completed parent trial, no patient or institution received personal or financial compensation. Ethical approval was obtained from the Bavarian Medical Chamber ethics committee (IRB Nr. 22091), and the trial was registered in the German Clinical Trial Register (DRKS00030880).

### Cases and intervention

The original intervention group (n = 14) of the parent trial were included for re-evaluation at a single time point at least six months after completing the 6-week FES program. A total of 10 (n = 10) patients participated in the FU. Two patients had died due to unrelated causes and another 2 patients declined further evaluation.

No further intervention was conducted after completion of the initial home exercise program and patients did not undergo any additional shoulder rehabilitation therapy before the FU visit. The assessed clinical outcomes therefore reflect the clinical FU after the previously completed 6-week FES-augmented home exercise program delivered in the parent trial.

All participants completed the standardized 6-week home exercise program for rTSA consisting of 12 progressive exercises performed 3 times per week to improve deltoid strength and mobility. The intervention group performed the program with the Shoulder Pacemaker (Shoulder Pacemaker, Alyve Medical, Inc) activated to deliver motion-triggered FES, while the control group trained with the device deactivated as a placebo.

### Clinical outcome measurements

Shoulder function was re-evaluated using the same methodology as described before. The primary outcome measure was the CS. Secondary outcomes included the SSV to assess patient-reported shoulder function and active ROM in abduction (Abd) ROM and forward flexion (Flex) ROM measured with a goniometer. Deltoid strength was quantified using a digital weight scale. In addition, total shoulder ROM and scapular kinematics were assessed using the automated ShowMotion system (NCS Company, Carpi, Italy). This system places motion sensors on the thorax, scapula, and upper limb to capture scapular translation, tilt, rotation, and shoulder girdle movement patterns. Scapulothoracic motion and muscle activity were then compared between the preintervention and FU evaluations.

### Statistics

Descriptive analyses were conducted for all primary and secondary outcome measures, including CS, SSV, ROM in Abd and Flex , deltoid strength, and scapular kinematics.

Continuous variables were given as mean and standard deviation, whereas categorical variables were presented as frequencies. Normality of data distribution was assessed using the Shapiro–Wilk test. For normally distributed variables, repeated-measures analysis of variance with Bonferroni post hoc correction was applied to compare preintervention, postintervention, and FU values. For non-normally distributed data, the Friedman test was used, and post hoc pairwise comparisons were performed using the Wilcoxon signed-rank test.

The Minimal Clinically Important Difference (MCID) in the parent trial for the CS was determined using a distribution-based approach and was set at MCID = 5.8 points. Given the small sample size in the FU cohort, distribution-based cohort-specific MCID estimation was not considered reliable. For interpretability, we applied a commonly used CS MCID threshold of 10 points when describing clinical relevance.

To assess the relationship between time to final FU and the CS at final FU, a Pearson correlation analysis was performed, as both variables were continuous and approximately normally distributed. Correlation coefficients (r) with corresponding two-tailed *P* values are reported. Given the small sample size, a Spearman rank coefficient (ρ) was additionally calculated as a sensitivity analysis.

Missing data resulted exclusively from patients lost to FU. Only participants who completed both the parent trial and the FU assessment were included in the final analysis of this study. No imputation methods were applied, and missing values were not replaced.

Statistical analyses were performed using Minitab 17 (Minitab, Inc., State College, PA, USA) and Jamovi (The jamovi project, version 2.6), with a significance threshold set at α < 0.05.

## Results

Fourteen participants comprised the original intervention group and 10 patients were available for long-term re-evaluation. Two participants had died of unrelated causes and 2 declined further assessment. The median interval between completion of the 6-week intervention and the long-term visit was 17.9 ± 3.54 months (range, 6-24 months). Baseline demographics and of the re-evaluated cohort are presented in Table I.

**Table I:** Baseline characteristics of the Follow-up group.

Baseline variable	Follow-up
Cases (n)	10
Age (yr)	79.6 ± 5.4
Right shoulder (n, %)	8 (80%)
Sex (female, n, %)	8 (80%)
Time to Follow-up after intervention (mo)	17.9 ± 3,54
Follow-up after surgery (mo)	42.8 ± 4,72

**Figure 1:**
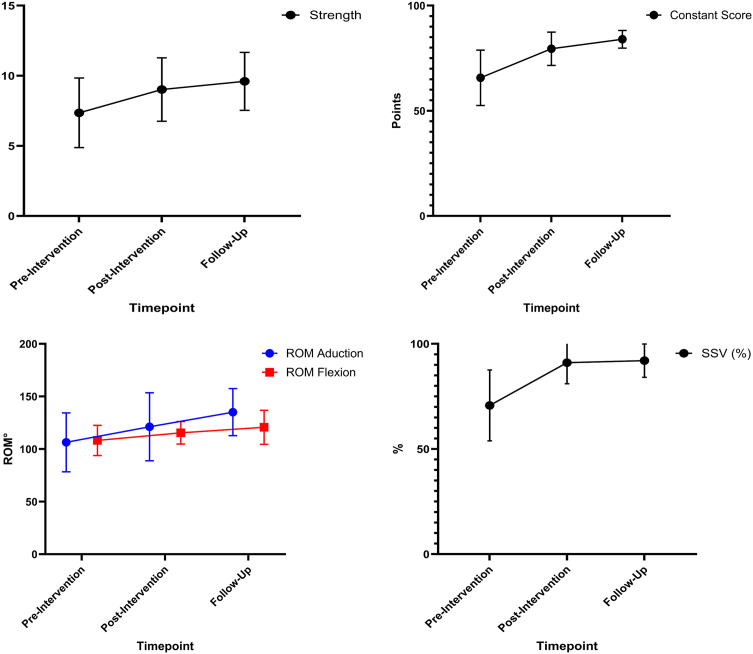
Time course of the clinical outcome parameters. Data are presented as mean and standard deviation. *SSV*, Shoulder Subjective Value; *ROM*, range of motion.

The mean CS improved from baseline (65.7 ± 13.2P) to immediate postintervention (79.5 ± 7.9P; percentage increase to baseline: +20,98%, *P* < .001). At long-term FU, the CS remained significantly higher relative to baseline (84.0 ± 4.2P; percentage increase to baseline: 27.83%, *P* < .001). with no significant change to the postintervention assessment (*P* = .093) (Fig. 1 and Tables II and III). No significant correlation was observed between time to final FU and CS at final FU (Pearson r = −0.21, *P* = .57; Spearman ρ = −0.16, *P* = .66). The proportion of participants exceeding the MCID of this study (>10 points) immediately post-intervention was 60% (6/10) and 70% (7/10) at long-term FU. The absence of a significant difference between postintervention and FU should not be interpreted as equivalence, particularly given the limited statistical power of this study.

**Table II:** Analysis of the intervention group pre-intervention, post-intervention and at follow-up.

Outcome variable	Preintervention	Postintervention	*P* value (pre vs. post)	Follow-up	*P* value (pre vs. FU)
Intervention group					
SSV (%)	70.7 ± 16.9	91.1 ± 10.0	**<.001**	92.0 ± 7.9	**<.001**
CS	65.7 ± 13.2	79.5 ± 7.9	**<.001**	84.0 ± 4.2	**<.001**
Strength	7.4 ± 2.5	9.0 ± 2.3	**<.001**	9.6 ± 2.1	**<.001**
Abduction (°)	106 ± 28	121 ± 32	.064	135 ± 22	**.004**
Flexion (°)	108 ± 14	115 ± 11	.149	121 ± 16	.059

*SSV*, Subjective Shoulder Value; *CS*, Constant Score; *FU*, follow-up.

Bold values indicate statistical significance at α = 0.05 (*P* < .05).

**Table III:** Comparison post-intervention and at follow-up.

Outcome variable	Postintervention	FU	*P* value (pre vs. post)
Intervention group			
SSV (%)	91 ± 10	92 ± 8	.704
CS	79.5 ± 7.9	84.0 ± 4.2	.093
CS (strength)	9.0 ± 2.3	9.6 ± 2.1	.328
Abduction (°)	121 ± 32	135 ± 22	.113
Flexion (°)	115 ± 11	121 ± 16	.407

*SSV*, Subjective Shoulder Value; *CS*, Constant Score; *FU*, follow-up.

The SSV increased from baseline (SSV = 70.7 ± 16.9%) to immediate postintervention (SSV = 91.1 ± 10.0%, percentage increase to baseline: +28.79%; *P* < .001) and was maintained at long-term FU (SSV = 92.00 ± 7.9%, percentage increase to baseline: 30,1%, *P* < .001). Similarly, there was no significant change in SSV between the postintervention assessment to the FU visit (*P* = .704).

Deltoid strength improved significantly after intervention (baseline strength: 7.36 ± 2.48P; postintervention strength: 9.02 ± 2.26P; percentage increase to baseline: +22.62%; *P* < .001) and remained significantly higher than baseline at lFU (FU strength: 9.60 ± 2.07P, *P* < .001). No significant change was seen in strength between postintervention and the FU (*P* = .328).

No significant improvement was seen in Abd ROM between pre- and postintervention evaluation (pre-Abd ROM = 106 ± 28° vs. post-Abd ROM = 121 ± 32°, *P* = .064) but there was a significant difference between baseline and the FU assessment (pre-Abd ROM = 106 ± 28° vs. FU-Abd ROM = 135 ± 22°, *P* = .004). However, the difference between the postintervention and FU value of Abd-ROM did not reach significance (*P* = .113).

No significant improvement was observed for forward Flex ROM between pre- to postintervention assessment (pre-Flex ROM = 108 ± 14° vs. post-Flex ROM = 115 ± 11°, *P* = .149), and there was no increase to the FU evaluation (pre-Flex ROM = 108 ± 14° vs. FU-Flex ROM = 121 ± 16°, *P* = .059).

Automated ShowMotion scapular kinematics continued to demonstrate no significant changes in scapulothoracic movement patterns at FU. No new device-related adverse events or late complications attributable to the FES intervention were observed.

## Discussion

This study reports a descriptive midterm FU of patients from the intervention arm of a prior randomized trial who completed an FES-augmented home exercise program after rTSA. In the original randomized controlled trial, patients treated with FES demonstrated significant improvements in CS, SSV, and active elevation, whereas the control group performing the identical exercise program without stimulation showed no significant changes.^20^ The current FU of the intervention cohort demonstrated that these gains were maintained, and no patient exhibited a decline in shoulder function. At FU, the improved outcomes observed immediately after the program remained above baseline in this cohort, and no participant demonstrated an obvious decline. Because the control group was not reassessed, it is not possible to determine whether maintenance is attributable to FES, exercise alone, or the natural post-operative course.

Most patients experience the greatest functional improvement within the first post-operative year after rTSA.^15^^,^^17^ Beyond this period, shoulder function typically reaches a plateau, with gradual decline reported in long-term FU.^15^^,^^17^ This late decline has been attributed to age-related changes rather than implant-related mechanical failure.^15^^,^^16^ While supervised and home-based rehabilitation is essential in the early post-operative phase after rTSA, emerging evidence suggests that standardized exercise programs may also provide additional functional gains beyond the initial recovery period.^18^^,^^20^^,^^21^ Uschok et al^18^ demonstrated that a structured physical therapy program in the mid- to long-term FU can further improve activities of daily living and ROM, although no significant gains in strength or pain reduction were observed. In this cohort, improvements across multiple parameters were maintained above baseline.

Successful shoulder movement after rTSA depends primarily on the deltoid muscle compensating for the deficient rotator cuff.^2^ Larger pre-operative deltoid cross-sectional area has been shown to correlate with superior post-operative strength and functional scores, whereas deltoid fatty infiltration negatively affects outcomes.^9^^,^^19^ These findings underscore the critical role of deltoid recruitment efficiency in functional performance after rTSA. The preserved functional results in this study cohort are consistent with, but do not prove, neuromuscular adaptation mechanisms.^1^^,^^3^

The sustained improvements observed may be explained by hypothesized neuromuscular and neuroplastic mechanisms.^1^^,^^3^ Repetitive neuromuscular electrical stimulation has been shown to enhance motor unit recruitment, increase corticospinal excitability, and reduce inhibitory drive, supporting the transition from externally facilitated contraction to improved voluntary.^1^^,^^3^ Akkaya et al^1^ demonstrated in their randomized controlled trial on rehabilitation after arthroscopic partial meniscectomy that electromyographic biofeedback and electrical stimulation accelerate recovery by improving voluntary muscle control. In a controlled laboratory study, Reinold et al^14^ reported acute gains in external rotation strength with neuromuscular stimulation, illustrating increased recruitment efficiency. However, potential mechanisms (eg, altered motor unit recruitment) remain speculative in the absence of objective neuromuscular assessments and should be addressed in future work.^1^^,^^3^^,^^14^

These findings justify further investigation in adequately powered comparative studies to determine whether motion-synchronized deltoid stimulation offers incremental benefit over structured rehabilitation alone in the late phase after rTSA. The intervention was well tolerated, home-based, and required no clinical supervision. Improvements were observed in this cohort beyond the first post-operative year. However, without a control group at FU, it remains unclear whether similar changes would occur with structured rehabilitation alone. Still, the result challenge the prevailing assumption that improvements cannot be expected once early recovery is complete.

The primary limitation of this study is the incomplete FU, with only 10 of the original 14 patients (71%) available for reevaluation. This introduces a potential risk of selection bias, as patients who participated in the FU may systematically differ from those lost, e.g., by having better functional outcomes, higher satisfaction, or greater engagement with rehabilitation. Consequently, the observed maintenance of functional improvements may overestimate the true effect within the original cohort. Although no formal comparison between included and lost patients was performed, this potential bias should be considered when interpreting the results. In addition, only the intervention group was re-evaluated, so potential spontaneous gains in the control group cannot be entirely excluded.

Furthermore, the relatively small sample size limits the generalizability of the findings and reduces the statistical power to detect smaller effect sizes, potentially increasing the fragility of some results.^22^ Moreover, the pilot trial was powered specifically to detect a clinically meaningful difference in the CS as the primary outcome measure. While secondary outcomes such as ROM, strength, and patient-reported scores were analyzed, the study was not powered to identify statistically significant differences in these variables.

Finally, the stimulation devices of the pilot trial and motion capture systems used in this study were provided by the manufacturer (Shoulder Pacemaker, Alyve Medical, Inc; ShowMotion, NCS Lab Srl, Carpi, Italy). However, the company had no role in the study design, protocol development, patient enrollment, data analysis, or manuscript preparation.

Despite these limitations, all procedures were conducted at a single high-volume center by one experienced surgeon ensuring consistency in surgical technique and post-operative management. The homogeneity of the study cohort strengthens the internal validity of the results. The results of this study with the demonstration of maintained improvements in CS, ROM, and strength following FES training in this small cohort supports the need for larger, adequately powered randomized controlled trials incorporating objective imaging or electrophysiological assessments to confirm structural and neuromotor adaptations.

## Conclusion

This prospective longitudinal FU study of the initial randomized controlled trial re-evaluated patients at least six months after completing a deltoid-focused FES-augmented home training program initiated 1 year after rTSA. The results demonstrated sustained improvements in shoulder function, ROM, and strength over time. These findings suggest that functional improvements may be maintained beyond the early recovery phase. However, due to the single-arm design, no conclusions regarding treatment-specific effects or modification of the recovery trajectory can be drawn. Larger, multicenter randomized controlled trials are needed to validate these outcomes and to optimize stimulation protocols and patient selection.

## Declaration of generative AI and AI-assisted technologies in the manuscript preparation process

During the preparation of this work, the authors used ChatGPT-5 (OpenAI Inc.) solely for language polishing, as none of the authors are native English speakers. Its use was strictly limited to stylistic and grammatical refinement. All content was subsequently reviewed and edited by the authors, who take full responsibility for the final published version.
